# *In vitro* and *in vivo* activity of 1,2,3,4,6-O-pentagalloyl-glucose against *Candida albicans*

**DOI:** 10.1128/aac.01775-24

**Published:** 2025-01-24

**Authors:** Lu Gao, Hao Wu, Jia Feng, Yu Liu, Ruina Wang, Lan Yan, Quanzhen Lv, Yuanying Jiang

**Affiliations:** 1 Department of Pharmacology, Shanghai Tenth People's Hospital, Tongji University School of Medicine 481875 , Shanghai, China; 2 School of Pharmacy, Naval Medical University 540408 , Shanghai, China; Providence Portland Medical Center, Portland, Oregon, USA

**Keywords:** 1,2,3,4,6-O-pentagalloyl-glucose, *Candida albicans*, invasive fungal infection, Eno1

## Abstract

Invasive fungal infections have become an increasingly serious threat to global human health, underscoring the urgent need for the development of new antifungal drugs. In this study, we found a natural polyphenolic compound 1,2,3,4,6-O-pentagalloyl-glucose (PGG), which is present in various plants and herbs. PGG showed broad-spectrum antifungal activities, enhancing the efficacy of fluconazole. Furthermore, PGG could protect mice against gastrointestinal and systemic infection with *Candida albicans*. Our mechanistic studies revealed that PGG exerts its antifungal effects partially by binding to the CaEno1 protein to inhibit its activity. As a crucial therapeutic target, Eno1 has been reported to be closely associated with cancer, hypertension, and infectious diseases. Our findings indicated that PGG, a new Eno1 inhibitor, is a potential candidate for further antifungal development.

## INTRODUCTION

Fungal infection is an important class of infectious diseases, encompassing both superficial and invasive infections (1). In recent years, the incidence of invasive fungal infections has notably increased due to the growing population of immunocompromised individuals (2). *Candida* species, particularly *C. albicans*, are the main cause of invasive fungal infections. Clinical invasive candidiasis could result in high mortality rates exceeding 70% (3). As an opportunistic pathogen, *C. albicans* commonly colonizes the human gut or superficial mucosa and can disseminate to vital organs by translocating through the intestinal barrier (4–6). This perturbation can lead to systemic infections when the microbiota is disturbed or immune defenses are compromised (7, 8). Due to the prominent role of intestinal *C. albicans* in causing candidemia, clearing *C. albicans* from the gastrointestinal tract may be a promising strategy to prevent invasive fungal infections (9, 10).

Dietary polyphenols are the most abundant bioactive compounds found in plants. Polyphenols are widely distributed in a variety of foods, including fruits, vegetables, cereals, tea, coffee, and wine. Consumption of polyphenols is beneficial to human gastrointestinal health (11). As a polyphenolic compound, pentagalloyl-glucose (PGG) is widely distributed in various plant-derived foods, such as fruits, gall nuts, and red wines (12). It has been identified as a highly bioavailable polyphenolic compound with remarkable antimicrobial activities. For example, PGG inhibited several bacterial strains including *Staphylococcus aureus*, *Staphylococcus epidermidis*, *Escherichia coli,* and *Pseudomonas aeruginosa,* and viruses including HSV, HBV, and HIV. Besides, it also exhibits other multiple functions such as anti-cancer, anti-oxidant, and anti-inflammatory activities (13–16).

By studying the effect of PGG on mammalian cells, Luo et al. found that PGG could inhibit glycolysis, a fundamental glucose metabolism pathway that occurs in the cytosol and generates ATP (adenosine triphosphate) and other metabolic intermediates. Glyceraldehyde 3-phosphate dehydrogenase (GAPDH) has been identified as the target of PGG (17). In our study, we found that PGG interacted with enolase 1 (Eno1), which catalyzes the production of phosphoenolpyruvate from 2-phospho-D-glycerate (2-PG) in glycolysis. The protein Eno1 plays a crucial role in the survival, pathogenicity, invasion, virulence, and antifungal drug susceptibility in *C. albicans* through its enolase and transglutaminase activities (18–22). Previously, we have reported that baicalein could exert its antifungal effects against *C. albicans* by targeting Eno1 and inhibiting glycolysis (23). Here, we found that PGG exhibited a higher binding affinity and inhibitory activity against *Ca*Eno1 compared to baicalein, providing a new lead for the development of Eno1 inhibitors.

In this study, we demonstrated that PGG has excellent in *vitro* efficacy against *Candida* and *Cryptococcus spp*. In the murine model of systemic and gastrointestinal *C. albicans* infection, PGG also showed protective effects when administered either orally or intraperitoneally. We have shown that PGG is bound to Eno1, resulting in inactivation of enolase activity and inhibition of *C. albicans* growth. Our study identified a new Eno1 inhibitor, PGG, which is meaningful for the development of antifungals.

## RESULTS

### PGG inhibited the proliferation of *Candida* species and *Cryptococcus neoformans*

**Fig 1:**
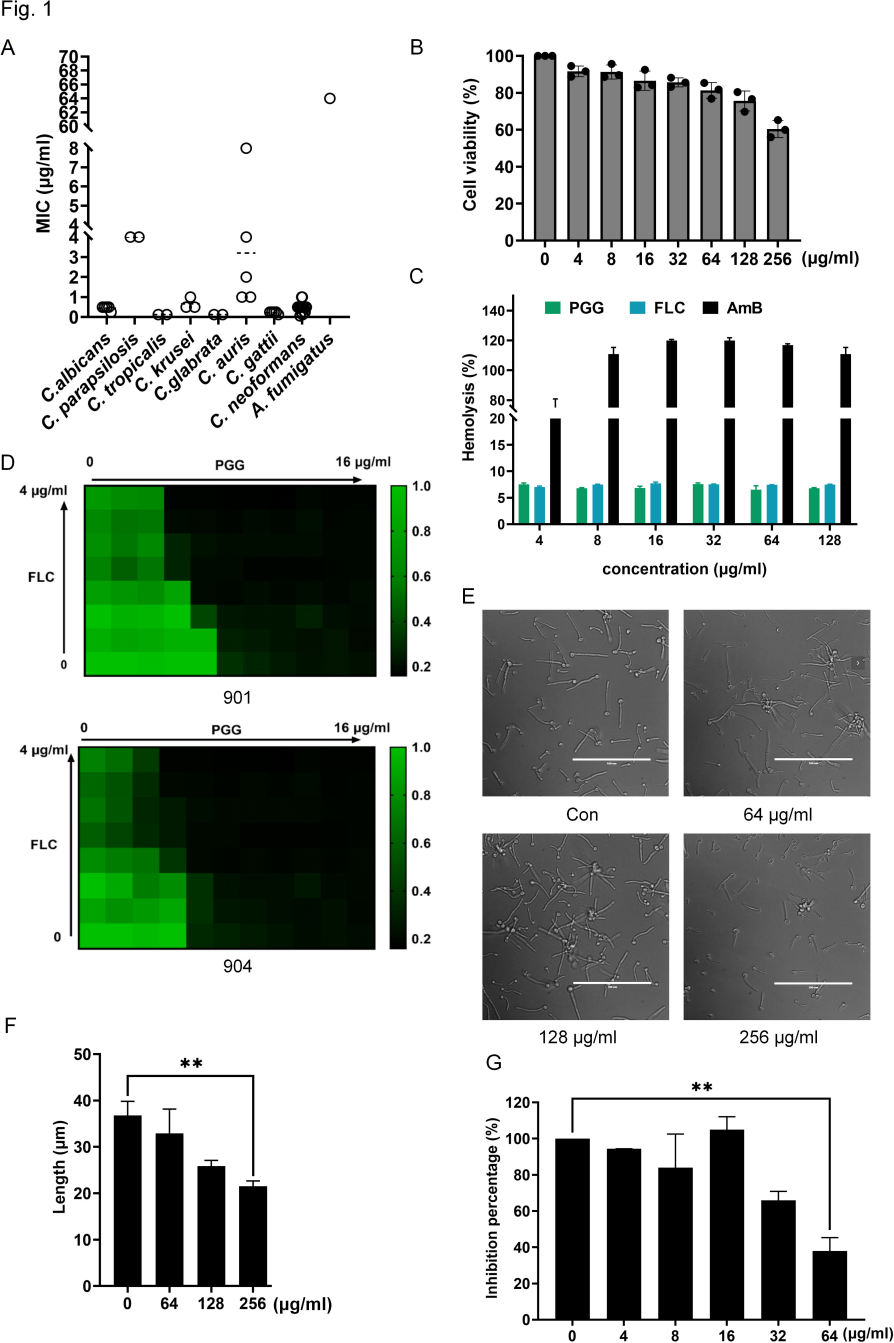
PGG shows potent *in vitro* efficacy against multiple fungal pathogens. (**A**) MICs of PGG against 42 fungal isolates. (*C. albicans n* = 5, *C. parapsilosis n* = 2, *C. tropicalis n* = 2, *C. krusei n* = 3, *C. glabrata n* = 2, *C. neoformans n* = 17, *C. gatti n* = 5, *C. auris n* = 5, and *Aspergillus fumigatus n* = 1). (**B**) Cytotoxic activity of PGG on HUVECs. Cell viability was performed by CCK-8 assay. (**C**) Changes in the inhibition rate of hemolysis. Values are averages of three experiments. (**D**) The synergistic antifungal effect between PGG and FLC against fluconazole-resistant *C. albicans* 901 and 904. (**E**) Inhibitory effect of compound PGG on hyphae formation. The hyphae formation of *C. albicans* SC5314 was induced in RPMI 1640 medium at 37°C for 3 h. (**F**) The length of hyphae was measured by ImageJ software. **P < 0.01, two-tailed unpaired t test. Each result was repeated two times. (**G**) Quantification of biofilms by XTT assay. Biofilm was incubated with XTT for 3 h at 37°C, and measured at OD_490_. **P < 0.01, one-way ANOVA. Each result was repeated at least two times.

The antifungal activity against fungal species was independently tested following the guidelines of the Clinical and Laboratory Standards Institute (CLSI). The minimum inhibitory concentration (MIC) of PGG is shown in Fig. 1A. Our results showed that the MICs against *Candida* species ranged from 0.125 to 8 μg/mL. Specifically, the MIC against *C. albicans* ranged from 0.25 to 0.5 μg/mL, against *Candida parapsilosis* was 4 µg/mL, *Candida tropicalis* was 0.125 µg/mL, *Candida krusei* was 0.5–1 μg/mL, *Candida glabrata* was 0.125 µg/mL. and against *Candida auris* was 1–8 μg/mL. Overall, PGG showed potent antifungal activity against *Candida spp*. In addition, PGG also showed strong activity against *Cryptococcus neoformans* with MICs ranging from 0.0625 to 0.5 μg/mL and *Cryptococcus gattii* ranging from 0.125 to 0.25 μg/mL. However, the inhibitory effect of PGG on *Aspergillus fumigatus* was not optimal, as indicated by a MIC of 64 µg/mL. The cytotoxicity of PGG was assessed using the CCK-8 assay, which was determined by the viability of human umbilical vein endothelial cells (HUVECs). At the concentration of 256 µg/mL, PGG reduced the proliferation of HUVECs by approximately 40%, indicating its remarkably low cytotoxicity (Fig. 1B). The selectivity index, defined as the ratio of IC_50_ against HUVECs to the MIC against *C. albicans*, is over 1,000-fold, also indicating a high level of specificity and safety for PGG (24). Meanwhile, the hemolytic activity of PGG was analyzed against rabbit red blood cells. PGG had no hemolytic or aggregative effect at a concentration of 128 µg/mL in the hemolysis test (Fig. 1C).

Azole resistance is common in *Candida* species. We also investigated the synergistic effect of PGG in combination with fluconazole against fluconazole-resistant *C. albicans*. When used alone, PGG at a concentration of 0.25–0.5 μg/mL significantly inhibited the proliferation of fluconazole-resistant *C. albicans* 901 and 904. When combined with 0.5 µg/mL fluconazole, the inhibitory concentration of PGG was reduced to 0.125 µg/mL (Fig. 1D). These findings suggest that PGG could potentiate the activity of fluconazole.

The inhibitory effect of PGG on *C. albicans* hyphae and biofilm formation was also investigated. Our results indicated that PGG had little impact on hyphae formation, with only a slight reduction in hyphal length observed at the concentration of 256 µg/mL (Fig. 1E and F). In line with this, using the 2,3-bis-(2-methoxy-4-nitro-5-sulfophenyl)−2H-tetrazolium-5-carboxanilide (XTT) assay, we found that 64 µg/mL of PGG could slightly inhibit biofilm formation, resulting in a decrease by 62.14% ± 5.28% relative to the control (Fig. 1G). In conclusion, the *in vitro* antifungal activity of PGG was robust, but its efficacy against hyphae and biofilms was limited.

### PGG protected mice against invasive *C. albicans* infection

**Fig 2:**
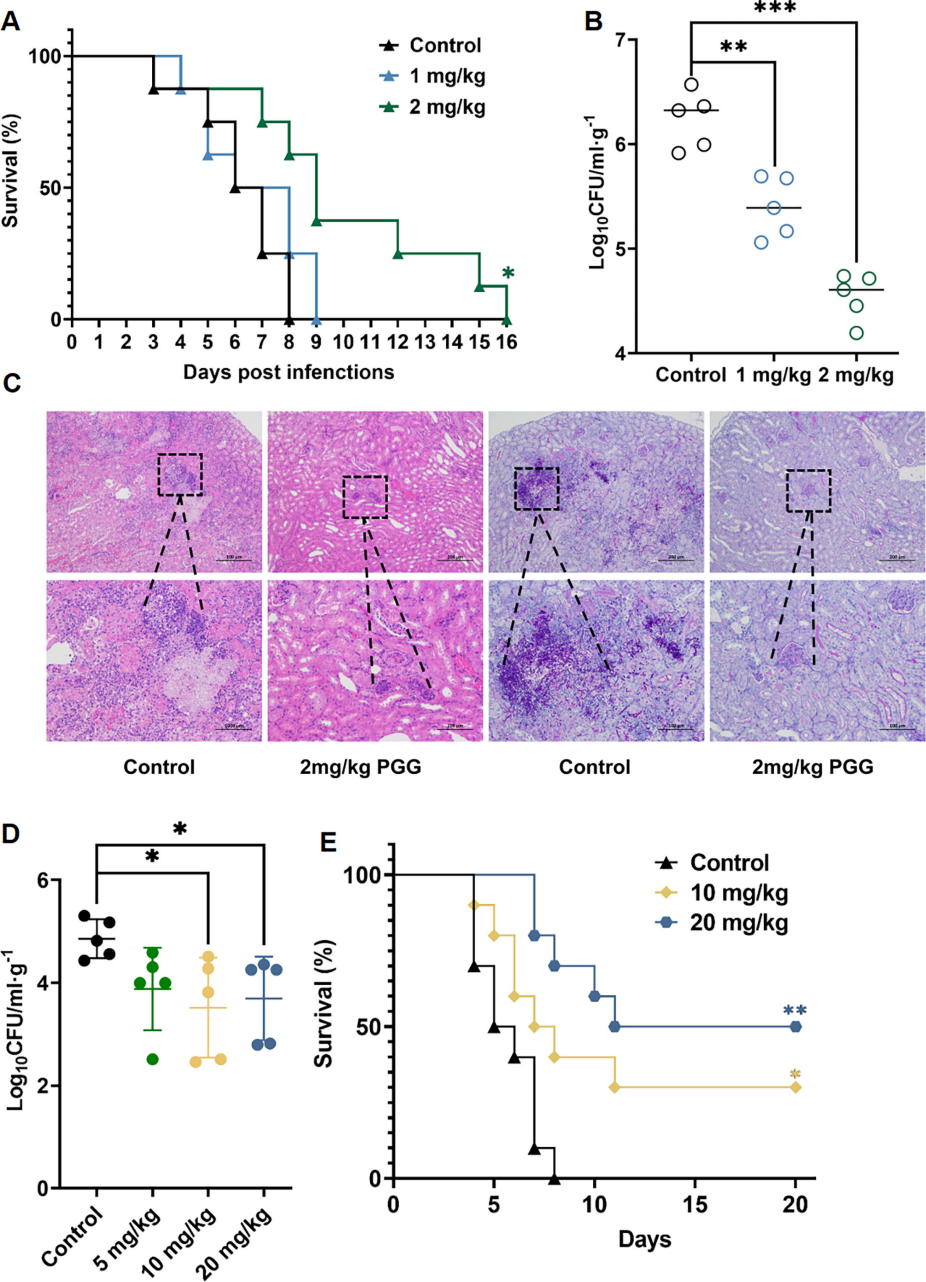
Efficacy of PGG in the mouse model of systematic *C. albicans* infection. (**A**) Survival of mice systemically infected with *C. albicans*. The female ICR mice were infected with 5 × 10^5^ CFU of *C. albicans* SC5314 through the tail vein and administered with 1 mg/kg or 2 mg/kg PGG intraperitoneally two times (*n* = 10). (**B**) Fungal burden in kidneys. (**C**) HE and PAS staining of the kidneys. The ICR mice were infected with 5 × 10^5^ CFU of *C. albicans* SC5314 through the tail vein and administered with 1 or 2 mg/kg PGG two times, and the kidneys were removed after 48 h of infection (*n* = 5). (**D**) Fungal burden in kidneys. The ICR mice were infected with 5 × 10^5^ CFU of *C. albicans* through the tail vein and administered with 5, 10, or 20 mg/kg PGG orally for times, and the kidneys were collected after 48 h of infection (*n* = 5). (**E**) Survival of mice systemically infected with *C. albicans*. The ICR mice were infected with 5 × 10^5^ CFU of *C. albicans* and administered with 10 mg/kg or 20 mg/kg PGG orally two times (*n* = 10). Two-tailed unpaired test (**B, D**), log-rank (Mantel-Cox) test (**A, E**). *P < 0.05, **P < 0.01, ***P < 0.001, ****P < 0.0001.

Models of gastrointestinal and systemic candidiasis, typically found in intensive care patients, post-abdominal surgery, or transplant recipients, have been used to evaluate the effect of PGG in mice (25, 26). The systemic *C. albicans* infection mouse model was established through tail vein injection, followed by intraperitoneal administration of PGG for treatment. The results showed that treatment with 1 mg/mL PGG did not improve the survival of infected mice. And 2 mg/kg of PGG did not increase survival rate but did increase the median survival time from 6.26 days to 10 days (Fig. 2A). At 48 h post-infection, treatment with 1 mg/mL and 2 mg/kg of PGG dose-dependently reduced colony-forming units (CFUs) in the kidney (Fig. 2B). Furthermore, the histological examination of the kidneys stained with hematoxylin-eosin (HE) and periodic acid-Schiff (PAS) showed that PGG treatment significantly reduced local inflammation and decreased the presence of *C. albicans* (Fig. 2C).

**Fig 3:**
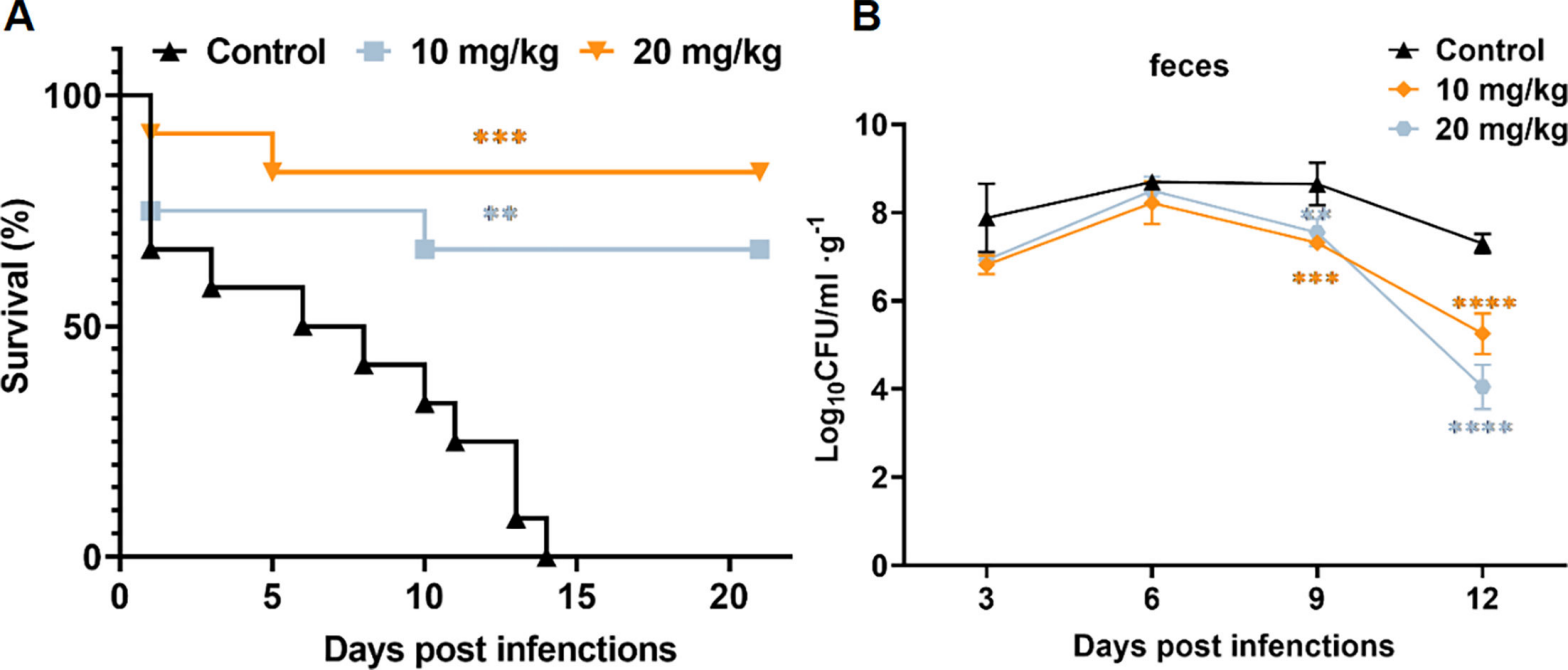
Efficacy of PGG in the mouse model of gastrointestinal *C. albicans* infection. (**A**) Survival of mice with *C. albicans* gastrointestinal infection. The female ICR mice were treated with cyclophosphamide (CTX) and levofloxacin, followed by infection with 4 × 10^8^ CFU/mice of SC5314 and treatment with 10 mg/kg or 20 mg/kg PGG orally once a day for 5 days (*n* = 10). (**B**) Fungal burden of feces in 3 days, 6 days, 9 days, and 12 days after gastrointestinal infection (*n* = 5). Data were shown as mean ± SD. Log-rank (Mantel-Cox) test (**A**), two-tailed unpaired test (**B**). *P < 0.05, **P < 0.01, ***P < 0.001, ****P < 0.0001.

It has been reported that oral administration of PGG can be used to treat diabetes, obesity, cancer, and other diseases, so we changed the route of administration to investigate its effect (27, 28). Due to the low bioavailability of oral administration, we increased the dose of PGG to 5, 10, and 20 mg/kg in the mouse model of systemic *C. albicans* infection. As shown in Fig. 2D, oral administration of 10 and 20 mg/kg PGG decreased the renal fungal load. Then, the doses of 10 and 20 mg/kg were selected to assess their impact on the survival rate. Our results showed that 10 and 20  mg/kg of PGG improved the survival rate of mice by 30% and 50%, respectively (Fig. 2E). In addition, a mouse model of gastrointestinal *C. albicans* infection was used to evaluate the antifungal efficacy of PGG via oral administration. Treatment with 10 and 20  mg/kg PGG improved the survival rates of mice to 60% and 80%. Meanwhile, the median survival time of PGG-treated mice was extended from 8 days to 14 days and 17 days (Fig. 3A). Consistently, the CFUs of fecal samples from PGG-treated mice were significantly reduced (Fig. 3B). At day 12 post-infection, the CFUs of PGG-treated mice exhibited a decrease of more than two orders of magnitude compared to the control group. Collectively, these results indicated that PGG exhibited a favorable protective effect against invasive *C. albicans* infection in *vivo*.

### PGG inhibits the enolase activity of *Ca*Eno1

**Fig 4:**
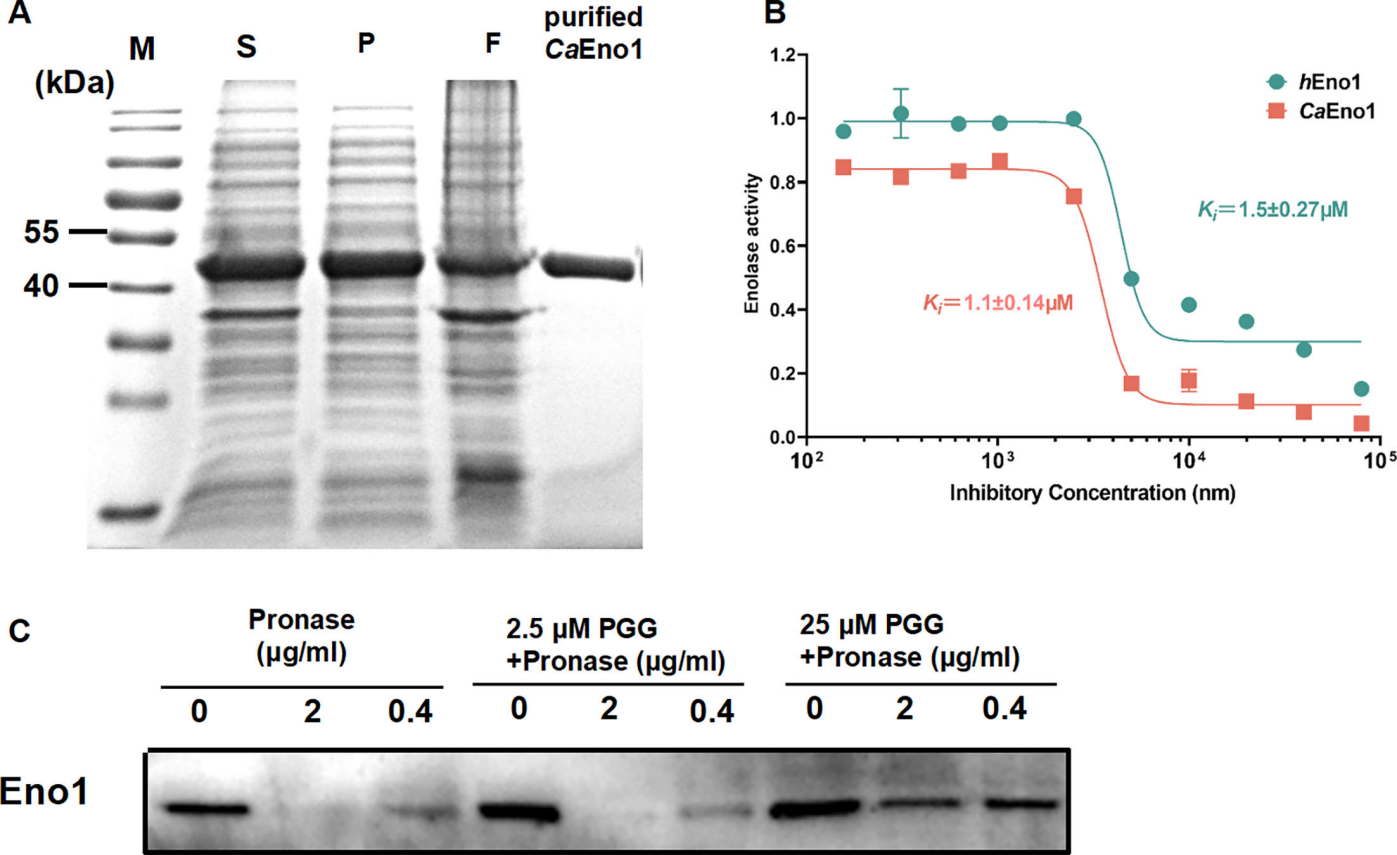
PGG binds specifically to Eno1 and inhibits its enolase activity. (**A**) Purification and identification of *Ca*Eno1 M: 10–180 KDa protein marker, S: Supernatant, P: precipitation, F: flow through. (**B**) The inhibition of PGG on the enolase activity. *Ca*Eno1 and *h*Eno1 were incubated with different concentrations of PGG. Data are shown as the means ± SD. (**C**) DARTS assay verified the binding between PGG and Eno1.

Our previous studies showed that baicalein, another type of polyphenol, can inhibit glycolysis in *C. albicans* by binding to Eno1 (23). Therefore, we speculated that the antifungal activity of PGG may also be related to Eno1. A reliable method for the expression of *Ca*Eno1 has been previously established (23). Purification was analyzed by SDS-PAGE and the results indicated that a protein of approximately 50 kDa was successfully obtained with a high degree of purification (Fig. 4A). To determine the effect of PGG on Eno1 function, the enolase activities of *Ca*Eno1 and hEno1 were tested. The inhibition constant (*K_i_*) of PGG in inhibiting *Ca*Eno1 is 1.1 ± 0.14 µM, which is lower than the IC_50_ of PGG in inhibiting hEno1, which is 1.5 ± 0.27 µM (Fig. 4B). To validate the selective inhibition on enolase, PGG was also incubated with horseradish peroxidase (HRP). Our results showed that PGG had no inhibitory effect on HRP below 12.5 µM (Fig. S1A). Then, to confirm the influence of PGG on the *Ca*Eno1, drug affinity responsive target stability (DARTS) analysis was performed, which demonstrated low degradation of *Ca*Eno1 treated with 25 µM PGG (Fig. 4C). Our results suggested that PGG could bind with Eno1 and inhibit its enolase activity, but the selectivity on *C. albicans* Eno1 is not obvious.

### *ENO1* gene dosage affects the sensitivity of *C. albicans* to PGG

**Fig 5:**
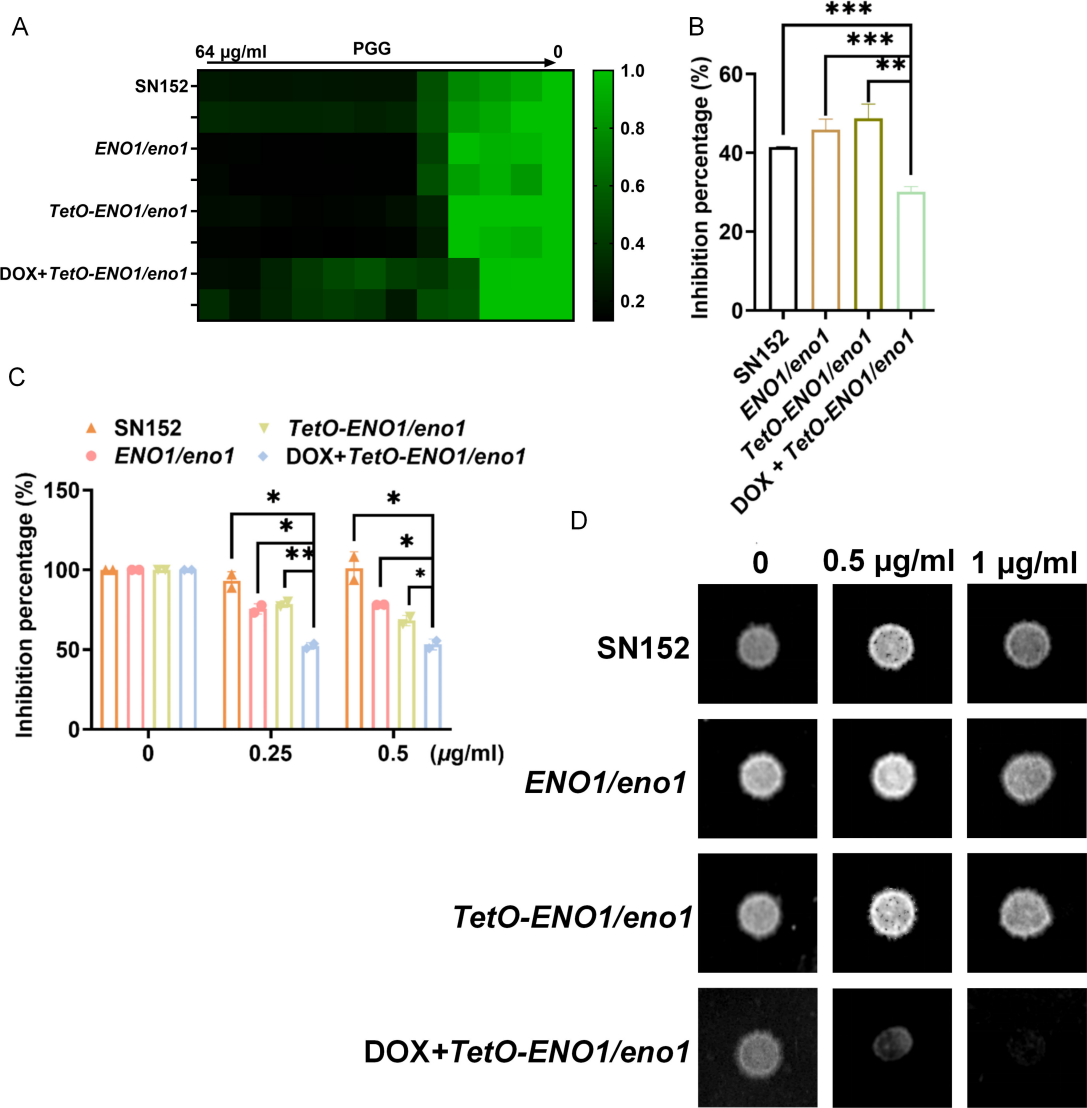
Downregulation of *ENO1* increases the sensitivity of *C. albicans* to PGG. (**A**) Growth inhibition of PGG. The growth of *C. albicans ENO1/eno1, TetO-ENO1/eno1, TetO-ENO1/eno1* with 100 µg/mL DOX was measured in the RPMI 1640 medium at 30°C for 48 h with or without PGG. (**B**) Inhibition percentage of PGG against *C. albicans* mutants determined by wet weight. The overnight cultured mutants were diluted to ~10^4^ CFU/mL with RPMI 1640 medium and treated with 1 µg/mL of PGG or 1 µg/mL of PGG plus 100 µg/mL DOX at 30°C for 24 h with shaking. (**C**) Growth inhibition of *C. albicans* determined by CCK-8 assay. The overnight cultured mutants were diluted to ~10^3^ CFU/mL and treated with 0.25 µg/mL of or 0.5 µg/mL of PGG at 30°C for 48 h. CCK-8 was incubated for 90 min at 37°C. (**D**) Spot assay. *C. albicans* mutants were grown on RPMI 1640 agar with or without PGG. Data were shown as mean ± SD. Two-tailed unpaired test (**B, C**). *P < 0.05, **P < 0.01, ***P < 0.001, ****P < 0.0001.

To further investigate the role of PGG on Eno1, the heterozygous deletion strain *ENO1*/*eno1* and the strain carrying the *ENO1* under the control of a tetracycline-regulated promoter were constructed (29). In the *TetO-ENO1/eno1* mutant, the expression of *ENO1* was suppressed by the presence of doxycycline (DOX). The addition of 100 µg/mL doxycycline decreased the mRNA level of *ENO1* by more than 10-fold, which was more significant than the *ENO1*/*eno1* (Fig. S1B). Meanwhile, the protein abundance decreased by about 30% (Fig. S1C). The growth of the different mutants showed that the heterozygous *ENO1/eno1* mutant was sensitive to PGG as same as SN152*,* but the *TetO-ENO1/eno1* mutant was more sensitive to PGG in the presence of DOX (Fig. 5A). In addition to quantifying the fungal cells using OD_600_, we also employed CCK-8 assay and measured wet weight of mutants cultured in RPMI1640. All these results confirmed that the *TetO-ENO1/eno1* mutant was more sensitive to PGG in RPMI1640 with DOX (Fig. 5B and C). In the solid medium, spot size was used to evaluate sensitivities. The density and size of the spot obviously decreased in the *TetO-ENO1/eno1* mutant when cultured in a medium containing PGG and DOX (Fig. 5D). The downregulation of *ENO1* results in increased sensitivity to PGG in *C. albicans*, suggesting that PGG exert its antifungal effects partially by inhibiting Eno1.

## DISCUSSION

Currently, natural polyphenols with diverse pharmacological properties and structures play a crucial role in drug discovery and development (30–33). Polyphenols are commonly classified into different groups, including phenolic acids, flavonoids, polyphenolic amides, and nonflavonoid polyphenols based on their chemical structures. Many polyphenols have shown antifungal activity, such as epigallocatechin, naringenin, quercetin, caffeic acid, and baicalein, with MICs ranging from 32 to >500 μg/mL (34). Our results indicated that PGG had higher antifungal activity compared to previously reported polyphenols. It has been reported that the MICs of PGG against *C. albicans* ranged from 0.5 to 1 µg/mL, against C. glabrata ranged from 0.25 to 0.5 µg/mL, against *C. parapsilosis* ranged from 0.5 to 4.0 µg/mL, and against *C. auris* ranged from 0.25 to 8.0 µg/mL. In this study, we found that PGG exhibited similar antifungal activity against *Candida* species in *vitro* compared with the published literature. In addition, PGG also showed antifungal activities against *C. krusei* (0.5–1 μg/mL), *C. neoformans* (0.0625–1 μg/mL), and *C. gattii* (0.125––0.25 μg/mL), which has not been reported before (16). More importantly, we further confirmed that PGG inhibited the growth of *C. albicans* in mice and protected the host in several models of *C. albicans* infection. Pharmacokinetic studies of PGG indicated that intraperitoneal injection of PGG achieved a blood concentration of 3–4 μM, which is approximately 11 times higher than the MIC of PGG (35). Oral administered PGG may be partially degraded, but it remains antifungal activity at higher doses (36).

Previous studies have shown that PGG’s antifungal activity is associated with iron chelation (16). However, chelation of PGG with iron may result in a structural change in PGG that diminishes its ability to bind to the target protein, rather than inhibiting fungal growth through intracellular iron deficiency. Furthermore, the previous study did not detect the changes in intracellular iron concentration and we propose that the antifungal mechanism of PGG was not limited to the iron chelation. Besides, it has been reported that PGG was a reversible inhibitor of GAPDH (17). The effective inhibitory concentration of PGG on the GAPDH of 293 T cells was about 6.7 µM (6.3 µg/mL), which was significantly higher than the MIC of PGG (0.5 µg/mL) against *C. albicans*. So, more effective targets may be responsible for the antifungal activity of PGG. In this study, our results suggested that PGG bound to Eno1 and inhibited the enolase of Eno1. As an essential gene, *ENO1* plays a crucial role in glycolysis. Eno1 can convert 2 PG to phosphoenolpyruvate and *ENO1*-disrupted *C. albicans* cannot survive in the medium with glucose alone. Eno1 also has transglutaminase activity and can bind to host plasminogen, enhancing the invasion and virulence in *C. albicans (*37). In addition to these functions, Eno1 is also involved in the process of gene transcription, translation, post-translational modification of proteins, and the host immune response of host to *C. albicans*. Disruption of *ENO1* makes *C. albicans* more susceptible to antifungal drugs such as amphotericin B, fluconazole, miconazole, and voriconazole. Consistently, our results suggested that PGG has synergistic or additive activity with fluconazole. The similar binding affinity of PGG to Eno1from *C. albicans* and humans raised the uninterpretable question about the activity in *vivo* and a low cytotoxicity of PGG. It was previously reported that among mammals, enolase exists in three isoenzyme forms, namely alpha-enolase, beta-enolase, and gamma-enolase, encoded by three different genes (38). Among those, α-Enolase has been implicated in a range of pathologies, encompassing infectious diseases, various malignancies, autoimmune disorders, and degenerative conditions. Therefore, the normal process of glycolysis can be maintained due to the redundant function among different Eno proteins when the activity of Eno1 was inhibited, which could explain the activity in *vivo* and a low cytotoxicity of PGG. This can also provide a putative speculation about the anticancer activity of PGG.

PGG is a more potent inhibitor of Eno1 than baicalein (23). The polyphenolic structure of PGG and baicalein may result in a similar antifungal mechanism. Here, we showed that PGG apparently inhibited the enolase activity and gene expression. To our surprise, the concentration of PGG that inhibited enolase activity was approximately 2 µg/mL, which is higher than the MIC of PGG at 0.5 µg/mL. This may be due to the limitations of the enolase enzyme activity assay, as Eno1 has many other functions. PGG may also affect the transglutaminase activity of Eno1 or act as an allosteric inhibitor of Eno1. The binding mode of PGG and Eno1 protein could be verified by molecular docking or eutectic interactions in the future. In addition, we found that the sensitivity of the *ENO1/eno1* mutant to PGG was not significantly increased compared to the parental strain SN152. This may be attributed to the high expression of the *ENO1* gene, which lacks the canonical TATA box and has many transcriptional start sites (39, 40). Meanwhile, it cannot be ruled out that Eno1 is only one of the proteins that interact with PGG, and other more efficient targets in *C. albicans* need to be further explored.

In summary, our study has demonstrated the potent in *vitro* antifungal activity of PGG. It was also shown to be effective in the murine gastrointestinal and systemic infection model. Mechanistically, PGG bound to Eno1 and inhibited its enolase activity, which will be a promising Eno1 inhibitor and antifungal lead compound.

## MATERIALS AND METHODS

### Strains, media, and reagents

*C. neoformans* strain H99 and ATCC 32609 and *C. albicans*, *C. krusei*, *C. tropicalis*, *C. glabrata*, *C. parapsilosis,* and *Aspergillus fumigatus* strains 7544 were from the fungal collection of our laboratory (41–43). *C. neoformans* clinical isolates (strains SCZ50100, SCZ50101, SCZ50102, SCZ50104, SCZ50106, SCZ50107, HN2-40, HN15, HN17, HN19, HN20, BJ3, BJ72, BJ95, and SH68), *C. gattii* strains WM178, WM179, E566, SCZ20024, and SCZ20031 were obtained from Shanghai Changzheng Hospital. The *ENO1* heterozygous deletion strain was constructed in a previous study (29). Prior to each in *vitro* and in *vivo* experiment, colonies of each strain were cultured in yeast extract-peptone-dextrose (YEPD) medium shaken overnight at 30°C. The cells were then centrifugated and washed in phosphate-buffered saline (PBS). The cell concentrations were determined using a hemocytometer. Fluconazole was purchased from Pfizer. Dulbecco’s modified Eagle’s medium (DMEM), cell counting kit-8 (CCK-8).

### *In vitro* susceptibility test

To determine the in *vitro* antimicrobial activity, a broth microdilution susceptibility assay was carried out in RPMI 1640 medium in accordance with the Clinical and Laboratory Standards Institute (CLSI) guidelines in CLSI document M27-A3 with few modifications. Fungal strains in RPMI 1640 medium (final concentration, ∼1  ×  10^3^ cells/mL) were prepared in 96-well plates, and antifungal agents were added. The final concentrations ranged from 0.0625 to 64  µg/mL for PGG. The plates were incubated at 30°C for 48 h (*C. albicans*, *C. krusei*, *C. tropicalis*, *C. glabrata*, and *C. parapsilosis*) or 72 h (*C. neoformans*, *A. fumigatus,* and *C. gattii*). The results were determined visually after incubation. The optical densities were subsequently measured using a Multiskan Sky (ThermoFisher Scientific, USA) at an absorbance of 600 nm. The MIC was defined as the lowest concentration of the compound that inhibited 90% or more of cell growth, as indicated by the OD_600_ compared to the control. All experiments were done in duplicate.

### Cytotoxicity test

HUVECs were used to evaluate the toxicity of PGG (44). HUVECs were diluted to 1  ×  10^5^ cells/mL in DMEM containing 10% fetal bovine serum (FBS), seeded in 96-well tissue culture plates, and incubated for 3 h for adhesion. After incubation, the supernatant was removed and fresh DMEM with different concentrations of PGG was added. The plates were incubated for an additional 24 h at 37°C with 5% CO2. Cytotoxicity was assessed by the CCK-8 assay (45). After incubation, 10 µL of CCK-8 solution (TargetMol Chemicals Inc.) was added to each well and the plates were incubated at 37°C for 2 h. Cell viability was assessed by measuring absorbance at 450  nm. Cells incubated in DMEM +FBS without PGG treatment were used as the standard for 100% viability. Three independent experiments were conducted.

### Hemolysis assay

Hemolysis experiments were performed with fresh rabbit red blood cells. Fluconazole and amphotericin B were used as positive control. All the compounds were tested at a concentration of 128 µg/mL. 4% rabbit red blood cells was obtained from Omash (Shanghai, China) Biotechnology Co. Ltd. The RBCs were centrifuged for 3 min at 5,000 rpm and then the supernatant was discarded. After being washed three times, the precipitates were re-suspended with equal volumes of PBS. 100 μL of RBCs per well was added into a 96-well plate. 200 µL of PBS was added into the first well. Compounds were diluted to 6.4 mg/mL and 2 µL added to the first well. After dilution, washed RBCs were incubated at 37°C for 1 h and then centrifuged. The supernatant was absorbed to another plate. The absorbance of 405 nm was determined.

### Hypha formation assay

The micro-dilution method was utilized to determine the minimum concentration of the compound that inhibited the growth of hyphae. The lowest concentration of the compound that prevented the visible growth of mycelium compared to the control was tested. RPMI 1640 medium containing approximately 1 × 10^4^ cells/mL of *C. albicans* was dispensed at 500 µL per well in 12-well plates. Subsequently, 10 µL of PGG serially diluted from 256 to 0.25 μg/mL were added to each well. After being cultivated at 37°C for 3 h, the hyphae were observed and captured by an EVOS microcopy. The hyphal length was measured using ImageJ software. Hyphae growth was measured in micrometers until the entire distance covered by the hyphae (linear *length*) was measured.

### Synergistic effect assay between PGG and fluconazole

The possible synergistic action of PGG in combination with fluconazole was determined by the checkerboard method. After cultured overnight, colonies of *C. albicans* were suspended in PBS and diluted to obtain a cell density of 1 × 10^3^ cells per 1 mL of suspension. PGG was established along the horizontal axis and fluconazole along the vertical axis. The eighth row contained only PGG and the twelfth column fluconazole. 100 µL of the prepared suspension was inoculated to each well of the plate. Plates were incubated for 48 h at 30°C. FICI (fractional inhibitory concentrations) was determined according to the equation FICI = FIC A + FIC B, where FIC A is the MIC of PGG in combination/MIC of PGG alone, FIC B is the MIC of fluconazole in combination/MIC of fluconazole alone. The combination is considered synergistic when the FICI is ≤0.5, indifferent when the FICI is >0.5 to <2, and antagonistic when the FICI is ≥2.

### Biofilm inhibition assay

The biofilm inhibition assay was performed as described (46). The XTT assay was utilized to quantify the density of the biofilms, and the percentage of biofilms inhibition was determined. *C. albicans* overnight cultures were re-suspended in the RPMI 1640 medium at a concentration of approximately 1 × 10^6^ cells/mL. Subsequently, 100  µL of the cell suspension was added to each well of a 96-well plate. Incubation of the plates at 37°C was conducted for 90 min for the anti-biofilm formation assay. After incubation, the wells underwent a single wash with PBS (pH 7.2–7.6) obtained from Sangon Biotech, Shanghai, China, to remove non-adherent cells. Subsequently, 100 µL of fresh RPMI 1640 medium, with or without PGG, was added in a serial dilution ranging from 64 to 0.125 μg/mL. The plates were then incubated at 37°C for 24  h. A dye solution was prepared by combining 9 mL of 0.5 mg/mL XTT with 1 mL of 0.32 mg/mL PMS (phenazine methyl sulfate) and allowed to stand for 15 min. Following incubation, the preceding medium in the 96-well plate was discarded, and the wells were gently rinsed three times with PBS. Subsequently, 100 µL of XTT-PMS staining solution was added to each well and incubated for 2 h in a lightless environment at 37°C. Finally, 75 µL of the supernatant from the 96-well plate was transferred to a fresh 96-well plate, and the optical density at a wavelength of 490 nm was determined using the Multiskan Sky instrument (ThermoFisher Scientific, Waltham, MA, USA).

### Mouse model of systemic infection with *C. albicans*

An established murine model of disseminated *C. albicans* with some modifications was used to evaluate the in *vivo* efficacy of PGG. ICR female mice weighing 18–22 g were used. To establish the model of systemic infection, mice were injected with 5  ×  10^6^ CFU of *C. albicans* SC5314 intravenously via the tail vein and then treated with PGG i.p. once a day for 2 days or p.o. once a day for 5 days.

### Mouse model of gastrointestinal infection with *C. albicans*

ICR female mice weighing 18–22 g were used in the study. To establish the model of systemic infection, mice were injected with 5  ×  10^6^ CFU of *C. albicans* SC5314 intravenously via the lateral tail vein then were treated with PGG I.p. or P.o.

### Mouse model of gastrointestinal infection with *C. albicans*

Female ICR mice of 28–31 g were purchased from Shanghai Regan Biotechnology Co., Ltd. Female ICR mice were maintained on drinking autoclaved water containing 5% glucose and antibiotics (levofloxacin hydrochloride, LEV, 0.4 g/L) throughout the experiments. Prior to the infection with *C. albicans*, mice were given an intraperitoneal injection of cyclophosphamide (CTX, 100 mg/kg) on two consecutive days. After oral administration of 4 × 10^8^ CFU of *C. albicans* for 2 h, the mice received an intraperitoneal injection of 100 mg/kg CTX and were treated with PGG orally. Then, the mice were injected intraperitoneally with 100 mg/kg of CTX at 3 and 6 days after infection and monitored for survival.

### Fungal burden

In the fungal burden assay, antifungal treatment was started 2 h after fungal inoculation and continued for 2 days. The day after termination of treatment, kidneys were harvested for quantitative determination of tissue fungal burdens. After determination of kidney weight, tissues were homogenized in 1 mL PBS. Homogenates were serially diluted 10-fold and aliquots (100 µL) of the homogenates were plated on SDA plates. The plates were incubated at 30°C for 48 h and the number of CFU was counted. Fungal loads were expressed as Log_10_ CFU/g. All experiments were carried out in duplicate.

### PAS and HE staining

PAS and HE staining was performed according to routine protocols. Briefly, after removal of the kidneys, tissue sections were fixed with 4% paraformaldehyde for more than 48 h and then stained by PAS or HE staining buffers (Wuhan Saiweier Biotechnology Co., Ltd). The pathological tissues were then examined and photographed.

### Expression and purification of recombinant *Ca*Eno1 and *h*Eno1

Specific protein acquisition details are described in a previous study from our laboratory (23). The plasmid pET-21a (1)-*ENO1* containing the *C. albicans ENO1*-coding sequence was transformed into *E. coli* strain BL21(DE3) (Tiangen, China). A single colony of BL21(DE3) transformed with the plasmid pET-21a (1)-*ENO1* was incubated in 50 mL of LB media containing 100 mg/mL ampicillin at 37°C for 14 h shaking at 200 rpm. The *E. coli* cultures were transferred into freshly prepared 1 L LB medium with 100 mg/mL ampicillin and incubated to an optical density at 600 nm (OD_600_) of 0.6–0.8. Then the culture was induced with 0.4 mM isopropyl-β-D-thiogalactopyranoside (IPTG), and the incubation was further carried out for 22 h at 16°C. Cells were harvested and then homogenized by sonication, and the lysate was centrifuged at 23,800 × *g* for 30 min at 4°C. The resulting supernatant containing the target protein fused with the 6 × His tag was further subjected to Ni-nitrilotriacetic acid (NTA) (Qiagen) affinity chromatography columns pre-equilibrated with lysis buffer and incubated for 30 min at 4°C. The columns were washed to remove contaminants, and the bound proteins were eluted and purified. SDS-PAGE assessed the peaks corresponding to target proteins. The recombinant hEno1 was expressed and purified using the same procedure as that of *Ca*Eno1(47).

### RT-qPCR

The reverse transcription of cDNA was performed with PrimeScript RT Master Mix (Perfect Real Time) with 500 ng of total RNA in 10 µL reaction mixtures. Each real-time quantification reaction mixture contained 1 µL of total cDNA, 0.8 µM gene-specific forward and reverse primers, 0.4 µL of ROX Reference Dye II (Merck Scientific), 10 µL SYBR Premix Ex Taq II(Merck Scientific), and 7 µL ddH_2_O in a final volume of 20 µL. As a control, actin gene transcript levels were determined with primers *ACT1-*F and *ACT1*-R. For the primers that were used for RT-qPCR, see Table S1. The thermal cycle parameters of RT-qPCR conditions were as follows: 2.5 min of enzyme preincubation at 95°C, followed by amplification for 60 cycles at 95°C for 20 s, 60°C for 34 s then melting at 95°C for 15 s, 65°C for 60 s, and 97°C for 1 s. The cycle threshold (*C_T_*) values of the *actin* control transcript were used to normalize the *C_T_* values of *Eno*1 transcripts. Each reaction was performed three times. The RT-qPCR data were analyzed by the 2^−ΔΔ*CT*^ method.

### Enolase activity analysis

Enolase activity was determined by direct spectrophotometric assay by measuring the increase of absorbance at 240 nm of phosphoenolpyruvate (PEP) as described previously with some modifications (23). Briefly, the reaction buffer (pH 7.0) containing 10 mM imidazole, 200 mM KCl, and 0.5 mM MgAc in a final reaction volume of 100 mL was mixed with Eno1 (a final concentration of 30 nM), followed by the mixture of 2-phosphoglycerate (2 PG) (Yuanye Biotech Shanghai) with a final concentration of 1 mM. The enolase activity of Eno1 was evaluated by measuring the increase in absorbance (OD_240_) at room temperature for 10 min. For the inhibition study of enolase by PGG, different concentrations of PGG were used to mix well with Eno1 and were incubated at room temperature for 5 min, and subsequent operations were described above. In the final reaction volume of 100 mL of this assay, the initial concentration of PGG was 8 mM and serially diluted twofold for 10 different concentrations. Inhibition curves were fitted by nonlinear regression (four parameters) using the GraphPad Prism software.

### DARTS assay

DARTS assay was used to assess the association of *Ca*ENO1 and PGG. *Ca*ENO1 was mixed with different concentrations of PGG for 1 h then supplemented with protease for 15 min at room temperature and centrifuged at 14,000 rpm for 15 min at 4°C. All steps were performed on ice to prevent premature protein degradation. To stop proteolysis, 1 × SDS loading buffer was added and heated as above immediately. Samples were loaded on SDS-PAGE. In the end, gels were stained with Western blot analysis.

### Western blotting analysis

Protein detection was performed by Western blot. SDS-PAGE electrophoresis was performed with equal amounts of protein. After being transferred to PVDF membranes, the blots were blocked with non-fat dried milk solution 5% (wt/vol) containing 5% skim milk powder at 30°C for 1 h. Afterward, they were incubated with primary antibodies 12G10 (1:5,000, Abcam) and Anti-Histone H3 antibody (1:2,000, Abcam) overnight at 4°C. After being washed with TBST, the HRP-binding secondary antibody was incubated at room temperature for 2 h. The protein bands were captured using the ECL detection system (Model 680, Bio-Rad Laboratories, Inc., Hercules, CA, United States) and analyzed with NIH ImageJ software (National Institutes of Health, Bethesda, MD, United States).

### Extraction of total protein from *C. albicans*

A single clone of *C. albicans* was inoculated in 1 mL YPD liquid medium at 200 rpm at 30°C for 16 h. Next, 1 mL of *C. albicans* overnight cultures was inoculated into 100 mL of fresh YPD liquid medium and placed onto a shaking table at 30°C at 200 rpm. They were cultured for 16 h and centrifuged at 4°C at 3,000 rpm for 5 min, the supernatant was discarded, and *C. albicans* cells were collected. The cells were washed three times with sterilized PBS (pH 7.4) and centrifuged at 3,000 rpm at 4°C for 5 min. A total of 10 mL of protein extract buffer (50 mm Tris, 1.5 mm EDTA, 1% Triton X-100, and 0.4% SDS [pH 7.5]) was added, and glass beads at two times the total volume were added. The mixture was transferred to the bead beater instrument and maintained on ice for 30 s at an interval of 30 s, and this procedure was repeated 10 times. The *C. albicans* cell lysates were removed and centrifuged at 13,000 rpm at 4°C for 5 min, and the supernatant was isolated and stored at −80°C.

### Wet weight determination

Overnight activated strains were diluted 10^4^-fold with fresh liquid RPMI 1640 medium in the presence of PGG or ddH_2_O. 50 mL diluted cultures were incubated at 30°C and 180 rpm for 24 h. The fungal suspension was collected using vacuum pump filters. Then, the wet weights were measured by using standard laboratory balances. The inhibitory percentages of PGG against strains were normalized to cells treated with ddH_2_O. The wet weight ratio between SN152 treated with PGG and SN152 treated with ddH_2_O (control) was calculated.

### Quantitative determination of cellular proliferation via CCK8 assay

Overnight strains were diluted to ~10^3^ then prepared in 96-well plates, and antifungal agents at 2 × concentrations were added. The final concentrations ranged from 0.0625 to 64 µg/mL for PGG. The plates were incubated at 30°C for 48 h. After incubation, 10 µL of CCK-8 solution was added to each well and the plates were incubated at 37°C for 1.5 h. 50 µL of supernatant was taken to measure absorbance at 450 nm. Cells incubated in RPMI 1640 without PGG treatment were used as the standard for 100% viability. Three independent experiments were conducted.

### Spot assay

*C. albicans* SN152, *Eno1/eno1,* and *TetO- Eno1/eno1* single colonies were incubated overnight in YPD medium at 30°C and 180 rpm. On the second day, the overnight cultures were diluted with sterile PBS (OD_600_ = 0.1). And, 1 µL of diluted fungal suspensions was spotted on RPMI 1640 agar or RPMI 1640 agar added with 100 µg/mL DOX in the presence of PGG or ddH_2_O. The agar was incubated at 30°C for 24–72 h. Finally, images were taken with a camera.

### Determination of horseradish peroxidase activity

HRP activity was used to detect the inhibitory selectivity of PGG. HRP enzyme was diluted 4,000-fold and incubated with different concentrations of PGG at room temperature for 15 min, 8 h, 24 h, and then we observed the inhibitory effect of PGG on HRP enzyme. HRP enzyme can catalyze the substrate TMB to generate products measured at 450 nm. PGG was diluted from 12.5 to 3.1 µM, Control incubations were conducted without PGG. The OD_450_ was measured at 10 min after the addition of substrate.

### Quantification and statistical analysis

Statistical analyses were performed using the analysis of two-tailed unpaired t test and Log-rank (Mantel-Cox) test through GraphPad Prism 9 program (GraphPad Software). All data were represented as means ± SD and were statistically considered when *P* values < 0.05 by selective statistical analysis methods, including data from animal experimentation and experiments in *vitro*. *P* values < 0.05 were classified into various significance levels: *P < 0.05, **P < 0.01, ***P < 0.001, ****P < 0.0001.

## SUPPLEMENTAL MATERIAL

Fig. S1 aac.01775-24-s0001.docx Gene and protein expression of ENO1 regulated by doxycycline.

Supplemental material aac.01775-24-s0002.docx Tables S1 and S2.

ASM does not own the copyrights to Supplemental Material that may be linked to, or accessed through, an article. The authors have granted ASM a non-exclusive, world-wide license to publish the Supplemental Material files. Please contact the corresponding author directly for reuse.
