# Supplementary material for: In vitro and in vivo activity of 1,2,3,4,6-O-pentagalloyl-glucose against Candida albicans
Source: Antimicrob Agents Chemother. 2025 Jan 24;69(3):e01775-24. doi: 10.1128/aac.01775-24 (PMC11881577; doi:10.1128/aac.01775-24)
Supplement: Fig. S1 — Gene and protein expression of ENO1 regulated by doxycycline. [file aac.01775-24-s0001.docx]

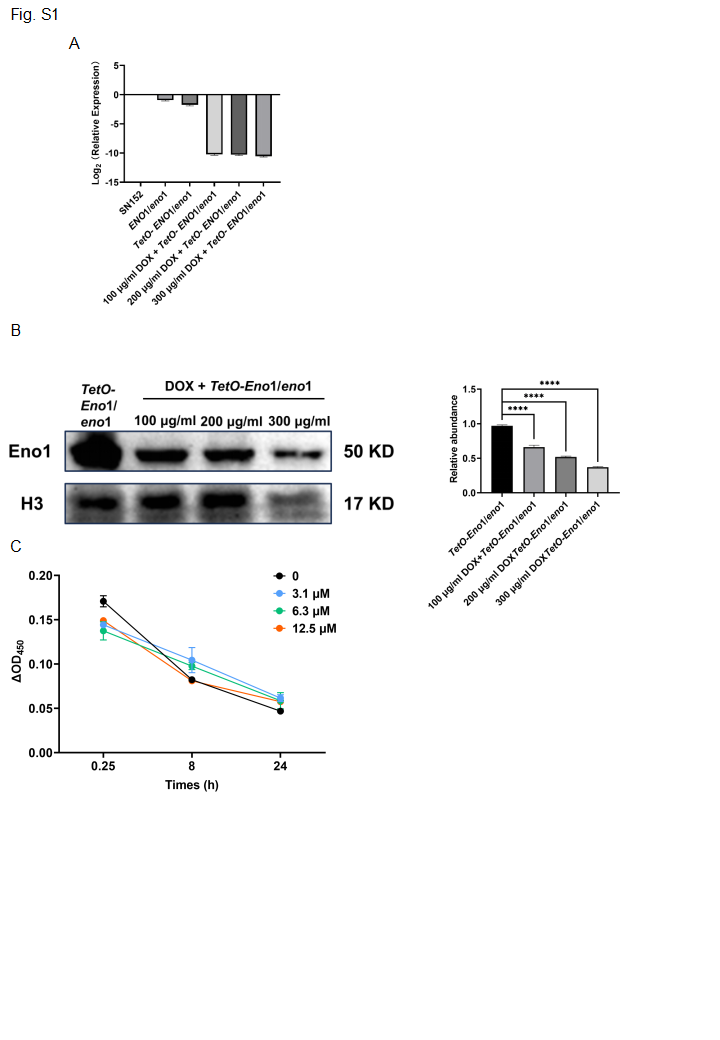
Fig. S1

(A) Real-time qPCR analysis for *ENO1* expression regulated by doxycycline after treated with doxycycline and incubated for 6 hours at 30 ℃. (B) Protein abundance of Eno1 expression regulated by doxycycline after treated with doxycycline and incubated for 6 hours at 30 ℃ then measured by western blot. (C) Absorption curves of TMB substrate catalyzed by HRP. HRP was co-incubated with PGG at room temperature after addition of TMB then ΔOD_450_ was measured at 15 min, 8 h and 24 h.
