# Supplementary material for: In vitro and in vivo activity of 1,2,3,4,6-O-pentagalloyl-glucose against Candida albicans
Source: Antimicrob Agents Chemother. 2025 Jan 24;69(3):e01775-24. doi: 10.1128/aac.01775-24 (PMC11881577; doi:10.1128/aac.01775-24)
Supplement: Supplemental material — Tables S1 and S2. [file aac.01775-24-s0002.docx]

| REAGENT or RESOURCE | SOURCE | GENOTYPE |
| --- | --- | --- |
| *C. albicans* and bacterial strains | | |
| *C. albicans* SC5314 | Lab stock | N/A |
| *C. albicans* SN152 | Lab stock | *arg4*Δ/ *arg4*Δ, *leu2*Δ/ *leu2*Δ, *his1*Δ/ *his1*Δ |
| *C. albicans Eno1/eno1* | Li W.et.al 2024 May^29^ | *HIS1, arg4/arg4, leu2 /leu2* |
| *C. albicans TetO-Eno1/eno1* | Li W.et.al 2024 May^29^ | *HIS1, ARG4, leu2/leu2* |
| *C. albicans* 901 | Lab stock | N/A |
| *C. albicans* 904 | Lab stock | N/A |
| BL21(DE3) | TIANGEN, China | F- ompT hsdSB(rB- mB-) gal dcm（DE3） |

Table. S1 Strains used in this study

(29) Li, W.; Feng, Y.; Feng, Z.; Wang, L.; Whiteway, M.; Lu, H.; Jiang, Y. Pitavastatin Calcium Confers Fungicidal Properties to Fluconazole by Inhibiting Ubiquinone Biosynthesis and Generating Reactive Oxygen Species. *Antioxidants* **2024**, *13* (6), 667. https://doi.org/10.3390/antiox13060667.

Table. S2 Primers used in this study

| No. | Primer name | Primer sequence (5’ to 3’) |
| --- | --- | --- |
| 1 | ENO1 up | GGCCATATGATGTCTTACGCCACTAAAATC |
| 2 | ENO1 down | CCGCTCGAGCAATTGAGAAGCCTTTTGG |
| 3 | ENO1-qF | GCTGCTAACGATTCTTACGCTG |
| 4 | ENO1-qR | ACCAGCGTAGATAGCTTCAG |
| 5 | ACT1-F | TTGATTTGGCTGGTAGAGAC |
| 6 | ACT1-R | ATGGCAGAAGATTGAGAAGA |
